# Movement Component Analysis of Reaching Strategies in Individuals With Stroke: Preliminary Study

**DOI:** 10.2196/50571

**Published:** 2023-12-05

**Authors:** Hirofumi Ota, Masahiko Mukaino, Yukari Inoue, Shoh Matsuura, Senju Yagi, Yoshikiyo Kanada, Eiichi Saitoh, Yohei Otaka

**Affiliations:** 1 Faculty of Rehabilitation School of Health Sciences Fujita Health University Toyoake Japan; 2 Department of Rehabilitation Medicine Hokkaido University Hospital Sapporo Japan; 3 Department of Rehabilitation Medicine I School of Medicine Fujita Health University Toyoake Japan; 4 Department of Rehabilitation Fujita Health University Hospital Toyoake Japan

**Keywords:** stroke, upper limb paresis, compensatory movements, three-dimensional motion analysis, reaching movement, rehabilitation, motion analysis, reaching, 3D, three dimensional, motion capture, motion, movement, limb, extremity, extremities, mobility, hemiparesis, paralysis, compensate, compensatory

## Abstract

**Background:**

Upper limb motor paresis is a major symptom of stroke, which limits activities of daily living and compromises the quality of life. Kinematic analysis offers an in-depth and objective means to evaluate poststroke upper limb paresis, with anticipation for its effective application in clinical settings.

**Objective:**

This study aims to compare the movement strategies of patients with hemiparesis due to stroke and healthy individuals in forward reach and hand-to-mouth reach, using a simple methodology designed to quantify the contribution of various movement components to the reaching action.

**Methods:**

A 3D motion analysis was conducted, using a simplified marker set (placed at the mandible, the seventh cervical vertebra, acromion, lateral epicondyle of the humerus, metacarpophalangeal [MP] joint of the index finger, and greater trochanter of the femur). For the forward reach task, we measured the distance the index finger’s MP joint traveled from its starting position to the forward target location on the anterior-posterior axis. For the hand-to-mouth reach task, the shortening of the vertical distance between the index finger MP joint and the position of the chin at the start of the measurement was measured. For both measurements, the contributions of relevant upper limb and trunk movements were calculated.

**Results:**

A total of 20 healthy individuals and 10 patients with stroke participated in this study. In the forward reach task, the contribution of shoulder or elbow flexion was significantly smaller in participants with stroke than in healthy participants (mean 52.5%, SD 24.5% vs mean 85.2%, SD 4.5%; *P*<.001), whereas the contribution of trunk flexion was significantly larger in stroke participants than in healthy participants (mean 34.0%, SD 28.5% vs mean 3.0%, SD 2.8%; *P*<.001). In the hand-to-mouth reach task, the contribution of shoulder or elbow flexion was significantly smaller in participants with stroke than in healthy participants (mean 71.8%, SD 23.7% vs mean 90.7%, SD 11.8%; *P*=.009), whereas shoulder girdle elevation and shoulder abduction were significantly larger in participants with stroke than in healthy participants (mean 10.5%, SD 5.7% vs mean 6.5%, SD 3.0%; *P*=.02 and mean 16.5%, SD 18.7% vs mean 3.0%, SD 10.4%; *P*=.02, respectively).

**Conclusions:**

Compared with healthy participants, participants with stroke achieved a significantly greater distance via trunk flexion in the forward reach task and shoulder abduction and shoulder girdle elevation in the hand-to-mouth reach task, both of these differences are regarded as compensatory movements. Understanding the characteristics of individual motor strategies, such as dependence on compensatory movements, may contribute to tailored goal setting in stroke rehabilitation.

## Introduction

Upper limb motor paresis due to a stroke may impose limitations on patients’ activities of daily living and quality of life [1-3]; the improvement of these conditions is an important goal of stroke rehabilitation. Therefore, periodic, accurate assessment of upper limb movement function during the course of treatment is fundamental to ensure effective upper limb retraining.

In rehabilitation settings, upper limb function is commonly assessed using several clinical measures, such as the Fugl-Meyer Assessment (FMA) scale, which assesses basic motor function [4], and the Action Research Arm Test, which assesses the basic functional capacity of the upper limb [5]. Previous studies have demonstrated the reliability and validity of these scales [6-8] and their clinical utility. However, clinical scales are based on clinician observation; thus, the risk of bias cannot be completely eliminated. In fact, the previously reported minimum detectable changes for these scales are large. Lin et al [9] reported that, according to interrater reliability, the minimum detectable changes of the FMA and Action Research Arm Test are 12.9 (20% of the total score) and 13.1 (23% of the total score), respectively. Such findings indicate that these clinical scales may have limited sensitivity to slight differences. In addition, these scales are used by summing the scores on the performance of various movements; therefore, they cannot be used to analyze a single movement in detail.

To address these issues, several studies have developed objective methods using measurement devices [10,11]. These efforts include movement tests with 3D motion analysis and robotic measurements, which have shown that various aspects of human upper limb motion can be quantified using these technologies [12-15]. Central to these analyses is the study of hand movements, as the hand serves as the primary end effector of the upper limb. What individuals can achieve with their upper limbs is determined by these hand displacements and their manipulation abilities.

Moreover, a better comprehension of altered body mechanics serves to guide clinical reasoning, develop evidence-based interventions, and monitor patients’ progress through follow-up [16]. In this regard, quantifying the underlying joint movement mechanisms that contribute to these upper limb movement patterns is essential for achieving better rehabilitation outcomes. Previous studies have shed light on the advantages and practicality of kinematic analysis for different reaching actions and real-world activities of daily living movements [17-22].

One of the major concerns in such kinematic analysis is the evaluation of compensatory joint movements. These movements serve as strategies to counteract the limitations posed by paresis to enhance the patient’s functional abilities. For those with severe paresis, reaching movements might be challenging. In such cases, interventions aim to boost compensatory actions, such as trunk flexion and shoulder abduction, to aid the movement [23,24]. However, these compensatory maneuvers usually consume more energy, making them less optimal than natural movements [25]. Conversely, in milder paresis, the intervention focuses on minimizing such compensatory motions to enhance movement efficiency [26,27]. In essence, although compensatory motions are essential when paresis is present, they should not be excessively relied upon when possible. Kinematic analysis offers a method to precisely quantify these movements. Clinically relevant activities, such as reaching and drinking movements, have been a focal point for kinematic studies. For example, Alt Murphy et al [17] highlighted the primary kinematic characteristics in patients with stroke during reaching and drinking movements. These features encompass compensatory actions, such as trunk tilt and shoulder abduction, as well as parameters, such as movement velocity and smoothness [17]. Aprile et al [28] reported the contribution of compensatory head motions while bringing a glass to one’s mouth. Since compensatory movements play a role in actions, such as reaching and drinking, it is crucial to assess their actual contribution. Quantifying this allows us to gain a deeper understanding of the functional movement challenges in patients with motor impairments, potentially streamlining the rehabilitation process by addressing core issues.

Drawing from these insights, we have devised a new analytical method to assess the impact of various movement components, including compensatory movements, on reaching actions. This approach quantifies and visualizes the contribution of each component during the reaching process. In designing our study, we prioritized clinical relevance, using a simplified methodology with a limited set of markers. Our goal in this study was to use this method to contrast the reaching strategies of patients with stroke against those of healthy individuals. We hypothesized that patients with stroke would exhibit a greater dependence on compensatory movements in reaching tasks compared to healthy individuals.

## Methods

### Participants

The participants were patients with hemiparesis due to stroke who underwent rehabilitation at Fujita Health University Hospital. The inclusion criteria were as follows: (1) existence of hemiparesis due to stroke with upper limb motor dysfunction, (2) ability to understand movement instructions, and (3) ability to maintain a sitting position for at least 30 minutes. The exclusion criterion was a history of neuromuscular or musculoskeletal diseases that could interfere with reaching movements of the upper limb.

In this study, healthy adults were included as the control group. Those with a history of neuromuscular or musculoskeletal diseases that might interfere with upper extremity reaching movements were excluded.

### Ethical Considerations

This study was approved by the institutional review board of Fujita Health University (HM21-006). Informed consent was obtained from all participants.

### Data Collection

A total of 2 upper limb reaching movements were measured in participants with stroke and healthy participants. While the 2 upper limb reaching movements in all healthy participants were measured using their dominant right hand, it was measured in patients with stroke using their affected side, irrespective of whether it was the dominant or nondominant side.

**Figure 1:**
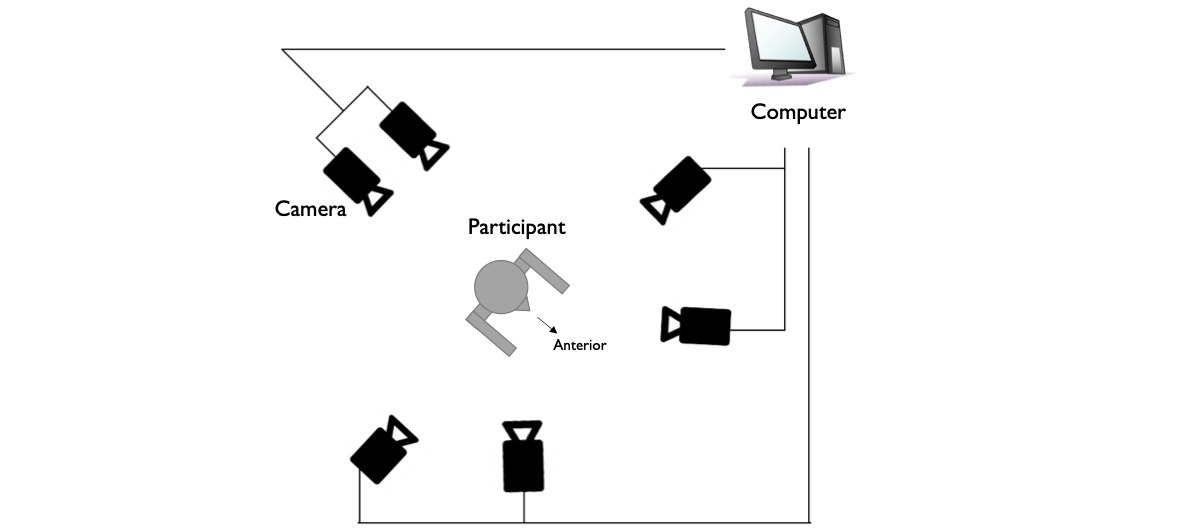
The setting of the 3D motion analysis system. The setup involves a computer responsible for data recording and analysis and 6 charge-coupled device cameras positioned around the participant.

**Figure 2:**
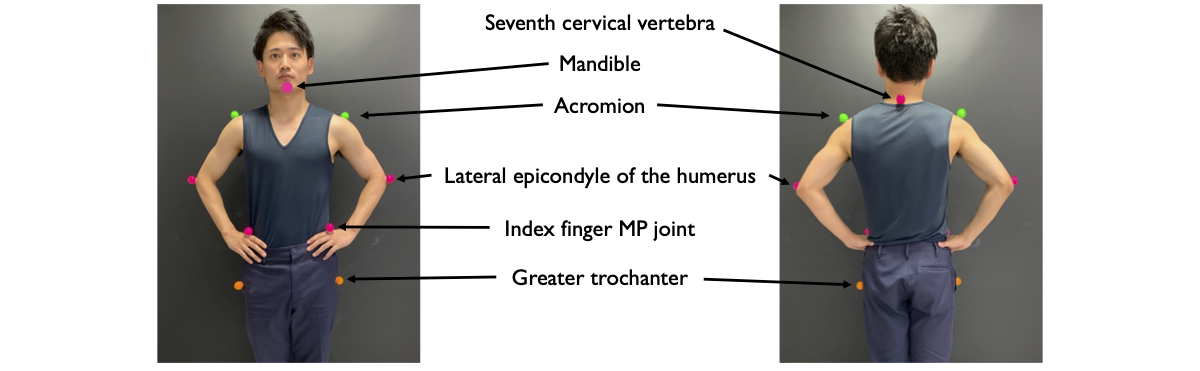
Marker locations. The markers were positioned at the following anatomical points: the mandible, the seventh cervical vertebra’s spinous process, the acromion, the lateral epicondyle of the humerus, the MP joint of the index finger, and the greater trochanter. MP: metacarpophalangeal.

A 3D motion analysis was conducted using KinemaTracer (Kissei Comtec Corporation), which comprises a computer that records and analyzes data and 6 charge-coupled device cameras installed around the participant (Figure 1). Measurements were made at a sampling frequency of 60 Hz. The 6 cameras were calibrated using control objects to minimize errors. The size of the control object was 120×60×50 cm. The average absolute error of the device was 1.5-2.4 mm on the left-right axis, 0.5-1.7 mm on the front-back axis, and 1.6-1.7 mm on the vertical axis, and is often used for motion analysis of the upper and lower limbs of participants with hemiplegia [29-31]. To develop a clinically feasible method to quantify the compensatory strategy, a simplified marker set was used. Colored markers with a diameter of 30 mm were affixed to the participants at 10 locations, namely, the mandible, the spinous process of the seventh cervical vertebra, the acromion, the lateral epicondyle of the humerus, the metacarpophalangeal (MP) joint of the index finger, and the greater trochanter (Figure 2).

During the measurements, the participants sat on a chair. During both the forward reaching and the hand-to-mouth reaching sessions, the participant’s hand was placed on a starting table set at a level of elbow and a distance of one-third of the upper limb length from the acromion in the upright trunk position. In both sessions, participants were instructed to carry an object that was 5.0 cm in diameter, 8.0 cm in length, and 50 g in weight at a comfortable speed. In the forward reaching session, participants were instructed to perform a reaching motion from the starting position to a platform placed at the maximum reaching position with the arm fully extended in an upright trunk position. In the hand-to-mouth reaching session, participants were instructed to bring their hands to their mouths from the starting position. All table heights were aligned with the forearm at 0° shoulder flexion and 90° elbow flexion.

In addition, we collected clinical findings: age, sex, and upper extremity length. Patients with stroke collected disease, postonset period, paralytic side, FMA total score of the upper extremity, and total score of shoulder, elbow, and forearm excluding reflexes.

### Data Analysis

The measured kinematic variables contained the 3D displacements (X-, Y-, and Z-axes) of the 10 markers. The X-, Y-, and Z-axes represent the lateral, forward or backward, and vertical directions, respectively.

**Figure 3:**
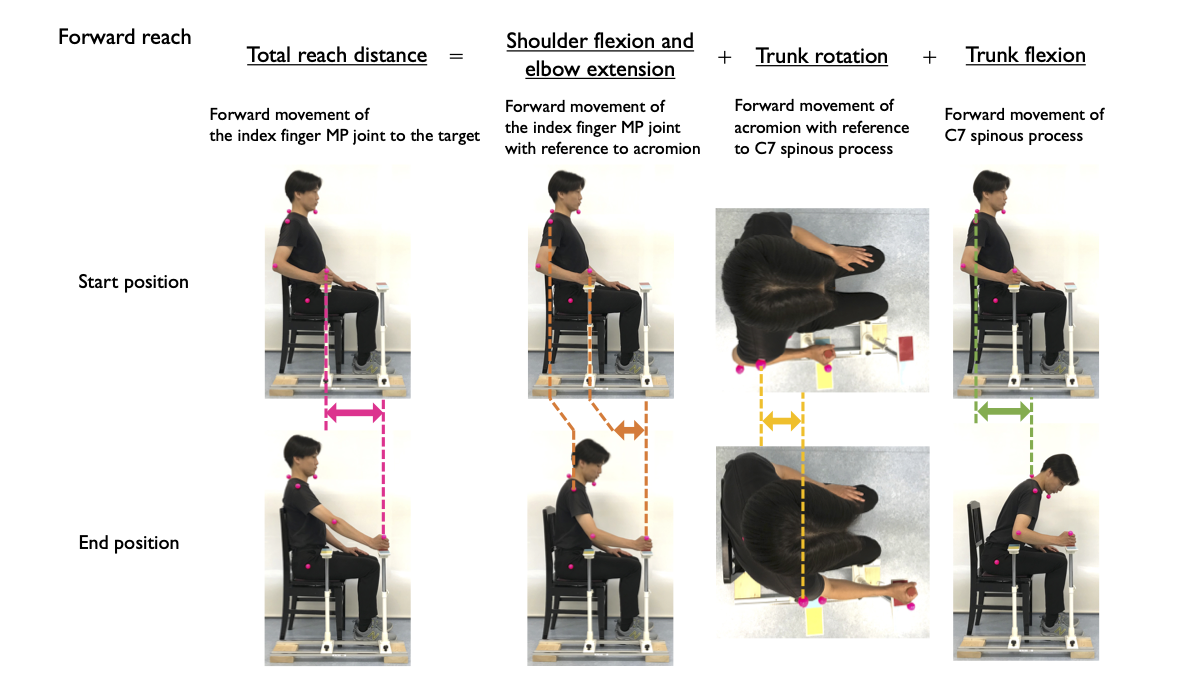
Components of the forward reach: shoulder flexion or elbow extension, trunk rotation, and trunk flexion. The distance in the forward-backward direction of each movement was calculated. MP: metacarpophalangeal.

For the forward reach task, the forward distance traveled by the finger MP joint from the starting position to the forward target location on the Y-axis was measured. Subsequently, the contributions of (1) shoulder flexion and elbow extension, (2) trunk rotation, and (3) trunk flexion toward the forward direction were calculated. The contribution of each movement was calculated as the forward movement of (1) the index finger MP joint with reference to the acromion, (2) the acromion with reference to the spinous process of the seventh cervical vertebra, and (3) the spinous process of the seventh cervical vertebra (Figure 3).

**Figure 4:**
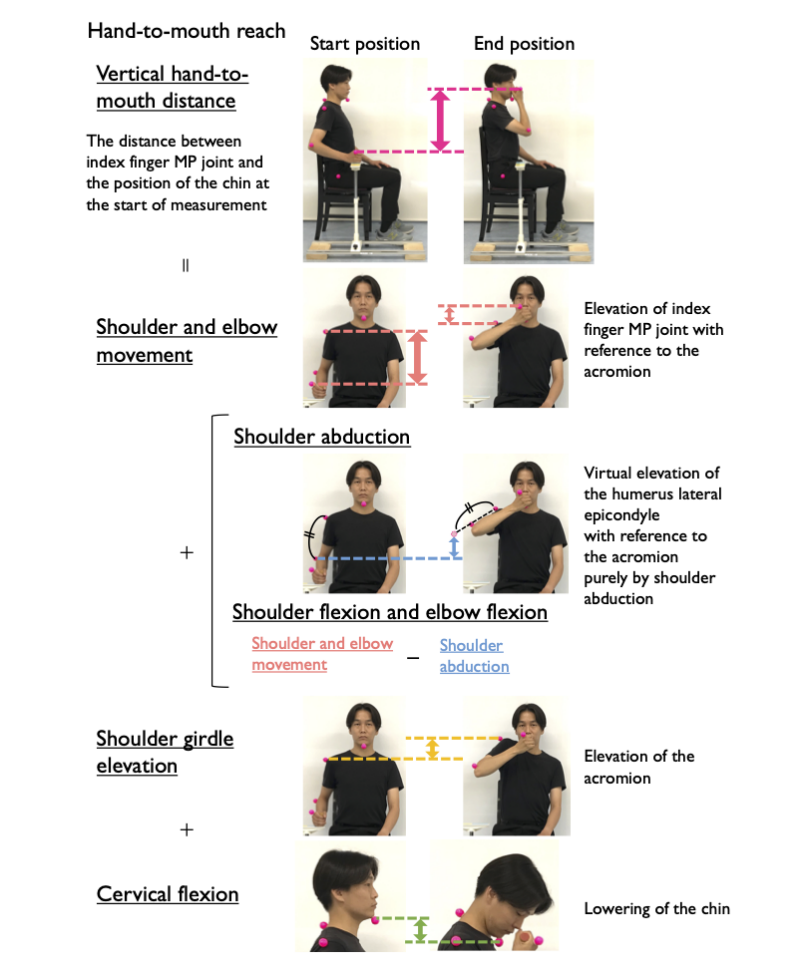
Components of hand-to-mouth reach: shoulder flexion or elbow flexion, shoulder abduction, shoulder girdle elevation, and cervical flexion. The distance in the vertical direction of each movement was calculated. MP: metacarpophalangeal.

For the hand-to-mouth reach task, the shortening of the vertical distance between the index finger MP joint and the position of the chin at the start of the measurement was measured. The contributions of (4) shoulder flexion and elbow flexion, (5) shoulder abduction, (6) shoulder girdle elevation, and (7) cervical flexion were calculated. The contributions of movements (4) and (5) were calculated in the following order: the sum of movements (4) and (5) (elevation of the hand through shoulder and elbow flexion) was calculated by the vertical displacement of the index MP joint with reference to the acromion, and movement (5) was calculated as the vertical elevation of the humeral epicondyle through shoulder abduction assuming the humeral epicondyle-acromion length did not change during abduction. The contributions of movements (6) and (7) were calculated as the vertical elevation of the acromion and the vertical drop of the chin, respectively (Figure 4). The displacement of each marker between the start and end positions in forward and hand-to-mouth reach tasks was calibrated by the position of the greater trochanter. The contribution of all components was calculated as a percentage of the total reach distance, which was determined by the arm length as mentioned above.

### Statistical Analysis

We performed a Student *t* test to compare each measure between participants with hemiplegia due to stroke and healthy controls to determine the characteristic movements of goal achievement in upper limb reaching movements for participants with hemiplegia due to stroke. All statistical analyses were performed using Statistical Package for Social Science (SPSS, version 28.0.1.0; IBM Corp), and the significance level was set at *P*<.05.

## Results

A total of 20 healthy individuals and 10 patients with stroke participated in this study. The demographic characteristics of the participants are presented in Table 1. The average age of healthy participants was 26.5 (SD 4.9) years, whereas that of participants with stroke was 70.7 (SD 13.8) years. Among the participants with stroke, 4 had intracerebral hemorrhage, and 6 had cerebral infarction. The average total score of the FMA was 52.1 (SD 10.7), and the subtotal of the components of the shoulder, elbow, and forearm, excluding reflex scores, was 23.9 (SD 5.3). All patients with stroke had left hemiparesis and thus were measured using their nondominant left hand, while all healthy individuals were measured using their dominant right hand.

**Table 1:** Demographic data of the participants.

			Healthy participants (N=20)		Stroke participants (N=10)
Age in years, mean (SD)			26.5 (4.9)		70.7 (13.8)
**Sex, n (%)**					
	Male	9 (45)		5 (50)	
	Female	11 (55)		5 (50)	
**Dominant hand, n (%)**					
	Right	19 (95)		10 (100)	
	Left	1 (5)		0	
Upper extremity length (cm), mean (SD)			71.7 (3.9)		69.4 (5.1)
**Diagnosis, n (%)**					
	Intracerebral hemorrhage	—^a^		4 (40)	
	Cerebral infarction	—		6 (60)	
Time after stroke (days), mean (SD)			—		76.6 (43.6)
**Affected side, n (%)**					
	Right	—		0	
	Left	—		10 (100)	
Fugl-Meyer Assessment (total score of upper extremity), mean (SD)			—		52.1 (10.7)
Fugl-Meyer Assessment (total score of shoulder, elbow, and forearm excluding reflexes), mean (SD)			—		23.9 (5.3)

^a^Not available.

**Figure 5:**
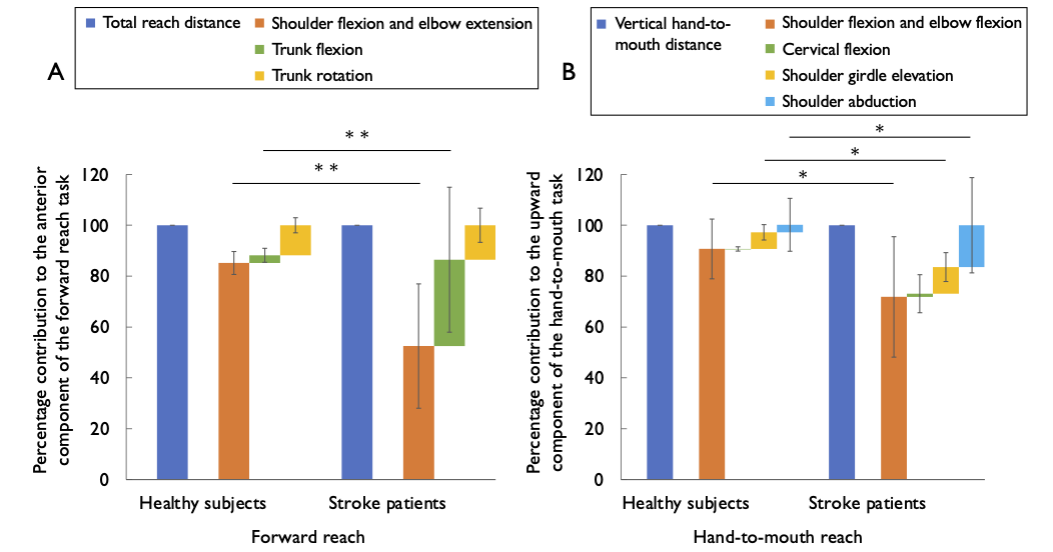
Quantification of dependence on compensatory movements. (A) Total reach distance in forward reaches on the Y-axis (blue) and its components in healthy participants and participants with stroke. Components: Shoulder flexion or elbow extension (orange), trunk flexion (green), and trunk rotation (yellow). (B) Vertical hand-to-mouth distance on the Z-axis (blue) and its components in healthy participants and participants with stroke. Components: Shoulder flexion or elbow extension (orange), cervical flexion (green), shoulder girdle elevation (yellow), and shoulder abduction (light blue).*P<.05, **P<.01.

The measurement results for the forward reach task and hand-to-mouth reach task are shown in Figure 5. All patients could reach the target in both the forward and hand-to-mouth reaching movements.

A comparison of the components of the forward reach revealed significant differences in the contribution of shoulder, elbow, and trunk flexion components between participants with stroke and healthy participants. The percentage contribution of shoulder and elbow flexion to the anterior component of the forward reach task was significantly smaller in participants with stroke than in healthy participants (mean 52.5%, SD 24.5% vs mean 85.2%, SD 4.5%; *P*<.001), whereas the percentage contribution of trunk flexion was significantly larger in stroke participants than in healthy participants (mean 34.0%, SD 28.5% vs mean 3.0%, SD 2.8%; *P*<.001). The percentage contribution of trunk rotation was not significantly different between participants with stroke and healthy participants (mean 13.6%, SD 6.7% vs mean 11.8%, SD 3.0%; *P*=.35).

A comparison of the components of the hand-to-mouth reach revealed significant differences in the contribution of shoulder and elbow flexion, shoulder girdle elevation, and shoulder abduction components between participants with stroke and healthy participants. The percentage contribution of shoulder and elbow flexion to the upward component of the hand-to-mouth task was significantly smaller in participants with stroke than in healthy participants (mean 71.8%, SD 23.7% vs mean 90.7%, SD 11.8%; *P*=.009), whereas the percentage contribution of shoulder girdle elevation and shoulder abduction were significantly larger in participants with stroke than in healthy participants (mean 10.5%, SD 5.7% vs mean 6.5%, SD 3.0%; *P*=.02 and mean 16.5%, SD 18.7% vs mean 3.0%, SD 10.4%; *P*=.02, respectively). The percentage contribution of cervical flexion was not significantly different between participants with stroke and healthy participants (mean 1.3%, SD 7.5% vs mean –0.2%, SD 0.9%; *P*=.42).

## Discussion

### Principal Findings

In this study, we attempted to analyze the reaching movement strategy in participants with stroke and healthy participants by quantifying the contribution of each movement component to forward and hand-to-mouth reaching. This study successfully identified that the movements that contributed to reaching differed between these populations. The contribution of shoulder flexion and elbow extension, which is the main component in the forward reach of healthy participants, was small in participants with stroke, whereas trunk flexion was significantly larger in participants with stroke than in healthy participants. In the hand-to-mouth reach, the contribution of shoulder and elbow flexion was smaller in participants with stroke than in healthy participants, whereas the contribution of upward elevation movements from shoulder abduction and shoulder girdle elevation was larger in participants with stroke than in healthy participants.

The difference in the movement strategies shown in this study may be attributed to movement abnormalities due to motor paresis of shoulder and elbow flexion and extension. In the participants with hemiparesis, the motor ability is retained in half of the body, and proximal muscles are relatively well restored during the course of recovery, possibly because of the bilateral innervation by the central nervous system [32]. Therefore, the impaired movement in the paretic side is often compensated by the nonparetic limb or proximal muscles with less severe paresis [33]. This analysis demonstrated that the contribution of a “normal” strategy in reaching, as quantified by the percentage contribution of shoulder flexion and elbow extension to the anterior component of the forward reach task, and the percentage contribution of shoulder and elbow flexion to the upward component of the hand-to-mouth task, was significantly reduced in reaching in participants with hemiparesis compared with healthy participants. These strategies, dominant in healthy participants, were replaced by compensatory joint movements not observed in the healthy participants: trunk flexion in the forward reach task and shoulder abduction and shoulder girdle elevation in the hand-to-mouth task. The findings of this study align with those of previous studies that highlight the role of compensatory trunk and shoulder movements [17,34,35]. Notably, Alt Murphy et al [17] demonstrated the relative contribution of such compensatory actions to reaching and drinking tasks. Building on this, our study delves deeper, quantitatively illustrating the contribution of each component to the functional objectives of reaching tasks.

### Clinical Implications

Recognizing the interrelationship of joint displacement and quantifying the degree of dependence, as in this pilot study, may be useful for clinical monitoring and goal setting of motor skills. In this study, participants with mild-to-moderate hemiparesis due to stroke were able to reach their primary target in reaching movement; however, they relied on compensatory movements to achieve this goal. Compensatory movements are generally considered inefficient compared to normal-like reaching movements, and reducing such compensatory movements seems to be an important goal of rehabilitation interventions in patients with this level of paresis. The quantification and visualization of the actual contribution of such compensatory movement should help clinical decision-making in planning interventions. Furthermore, the visualization of the degree of dependence on compensatory movements may be more useful when combined with the indexing of skill goal achievements—in this case, the reach distance—to measure the effectiveness of interventions. When analyzing the effects of interventions or assistive devices, examining the motor skills essential for achieving goals, such as forward reach task and hand-to-mouth task, along with the reliance on compensatory movements, can offer a deeper insight into how an intervention might impact patient activity.

### Limitations

This study had several limitations. First, the measurement did not include the speed of each reaching performance. Considering that the speed of movement tasks affects other aspects of performance, this may have influenced the results of this study. A more detailed analysis in the future with additional performance indices, such as speed, smoothness, and accuracy, could improve the understanding of the reaching ability of patients with motor paresis. Furthermore, the limited number of markers in this study prioritizes clinical application and ease of implementation. However, this constraint poses challenges in analyzing additional parameters like joint angles. Balancing this focus on contribution data with clinical feasibility requires further deliberation on the need for more detailed information. Second, the sample size was limited, and there was a significant variation in the index values. All patients had left hemiparesis, which meant measurements were performed using their nondominant hands. In contrast, the dominant right hand was used for the healthy participants. Robertson et al [36] pointed out that there might be performance differences between left and right paresis, which could further influence the study outcomes. The cumulative impact of these biases might affect the results. It is also worth noting that the control group comprised young adults, which could restrict the accuracy of gauging the impact of hemiparesis in this study. Despite this limitation, we believe our results retain their significance by effectively visualizing the unique motor strategies that patients with stroke use during reaching actions, emphasizing the crucial role of compensatory movements. Future studies, ideally with larger sample sizes and age- and side-matched controls, will certainly further refine our understanding of motor impairments in reaching. Despite the acknowledged limitations, this study provides meaningful contributions by introducing a simplified method to quantify and visualize joint motion strategies for reaching movements, thereby potentially facilitating the clinical application of upper limb motion analysis.

### Conclusions

In this study, our goal was to assess the contributions of various joint movements to reaching actions and to distinguish between patients with stroke and healthy participants. Our analysis successfully highlighted a distinctive motor strategy in patients with stroke: the typical contributions of shoulder and elbow joint movements seen in healthy individuals were diminished, whereas the contribution of other joints, often deemed compensatory movements, was elevated in patients with stroke. The detailed evaluation of reaching ability this study offers, considering both actual reaching proficiency and reliance on compensatory movements, provides a valuable foundation for setting pertinent and effective rehabilitation goals.

## Abbreviations

FMA: Fugl-Meyer Assessment
MP: metacarpophalangeal

## Data Availability

The data collected and analyzed during the study are available from the corresponding author upon reasonable request.
